# Evaluating the Diagnostic and Treatment Capabilities of GPT-4 Vision in Dermatology: A Pilot Study

**DOI:** 10.1177/12034754251336238

**Published:** 2025-05-06

**Authors:** Abhinav Pillai, Sharon Parappally-Joseph, Jason Kreutz, Danya Traboulsi, Maharshi Gandhi, Jori Hardin

**Affiliations:** 1 Division of Dermatology, Department of Medicine, Cumming School of Medicine, University of Calgary, Calgary, AB, Canada

**Keywords:** artificial intelligence, dermatology, GPT-4V, diagnostic accuracy, treatment planning

## Abstract

**Background:**

The integration of generative artificial intelligence within dermatology presents a new frontier for enhancing diagnostic accuracy and treatment planning.

**Objective:**

This research evaluates Generative Pre-trained Transformer-4 Vision’s (GPT-4V) performance in accurately diagnosing and generating treatment plans for common dermatological conditions, comparing its assessment of textual versus image data and its performance with multimodal inputs.

**Methods:**

A dataset of 102 images representing 9 common dermatological conditions was compiled from dermatlas.org and dermnet.nz. Images were screened by 2 board-certified dermatologists and were excluded if they did not represent a classic presentation of the respective conditions. Fifty-four images were included in the final analysis. In addition, 9 text-based clinical scenarios corresponding to each condition were developed. GPT-4V’s diagnostic capabilities were assessed across 3 setups: Image Prompt, Scenario Prompt, and Image + Scenario Prompt.

**Results:**

In the Image Prompt setup, GPT-4V correctly identified the primary diagnosis for 54% of the images. The Scenario Prompt and the Image + Scenario Prompt setups, respectively, both achieved an 89% accuracy rate in identifying the primary diagnosis. Treatment recommendations were evaluated using a modified Entrustment Scale, showing competent but not expert-level performance. A Wilcoxon signed-rank test demonstrated a statistically significant difference in treatment recommendations based on the Entrustment Score, with the model performing better in the Image + Scenario setup (*P* < .01).

**Conclusion:**

GPT-4V demonstrates the potential to augment dermatological diagnosis and treatment recommendations, particularly in text-based scenarios. However, its underwhelming performance in image-based diagnosis and integration of multimodal data highlights important areas for improvement.

## Introduction

Generative artificial intelligence (AI), notably Generative Pre-trained Transformer (GPT) models like ChatGPT, has garnered widespread attention for its ability to understand and generate human-like responses.^1,2^ The launch of GPT-4 Vision (GPT-4V), a multimodal large language model (LLM) capable of processing both image and text inputs, represents another significant milestone in AI.^1,2^ This development is of particular relevance to dermatology, a field heavily reliant on visual information for diagnostics and treatment planning.

Recent literature highlights the progress of GPT models in dermatology.^3,4^ Kluger highlighted the potential applications of ChatGPT in dermatology, especially in accurate disease identification and differential diagnosis.^4^ Generative AI has also been employed to assist in cutaneous malignancy classification.^5^ ChatGPT specifically may help with triaging cutaneous neoplasms, although improvements are needed in specialized areas like Mohs surgery triaging.^6^ Another area where generative AI has demonstrated potential is in teledermatology, where a GPT-based chatbot was used to aid in the teledermatology consultation process.^7^ AI has also demonstrated notable potential in dermatopathology, with the potential for convolutional neural networks to perform at greater sensitivity and specificity levels than dermatopathologists.^8^ Beyond clinical decision-making in dermatology, AI can assist with literature searches, medical education, patient education, identifying patterns from electronic health records, and direct-to-consumer mobile phone apps.^9,10^

Despite the potential of AI in dermatology, significant gaps remain. For example, evaluations of earlier GPT models on dermatology questions from the United States Medical Licensing Examination (USMLE) were limited by the AI’s lack of vision capabilities, preventing image analysis.^11,12^ GPT-4V’s vision capabilities may overcome this limitation,^1,2^ enabling more accurate and comprehensive dermatological assessments.

While ChatGPT has demonstrated some proficiency in handling patient queries in dermatology,^13^ GPT-4V’s ability to process both text and image inputs presents a greater opportunity for LLMs to augment dermatological assessments. However, it is unclear how GPT-4V performs in diagnosing dermatological conditions when compared to earlier models, particularly in integrating multimodal inputs. There is also a need to address its limitations in contextualizing complex cases and providing nuanced recommendations in specialized areas.

This research evaluates the performance of GPT-4V at accurately diagnosing and formulating treatment plans for common dermatological conditions. Specifically, it compares the model’s performance in interpreting textual versus image data and its performance in integrating multimodal inputs, combining text and image data. In addition, this work evaluates the broader trajectory of integrating AI into the clinical practice of dermatology.

## Methods

### Image Collection

A dataset of 102 images with predetermined diagnoses was compiled from publicly available sources, specifically dermnet.nz and dermatlas.org. The images were obtained from open-access platforms to ensure compliance with Canadian copyright laws and privacy regulations while still contributing to a robust evaluation of GPT-4V. The selected images comprised 9 common dermatological conditions, including acne vulgaris, rosacea, atopic dermatitis (AD), psoriasis, actinic keratosis, basal cell carcinoma, squamous cell carcinoma (SCC), superficial spreading melanoma (SSM), and vitiligo. These conditions were selected for their clinical prevalence and the availability of authentic, deidentified images from open-access sources.

The accuracy and quality of the images were evaluated by 2 board-certified dermatologists (JH and DT). Given that an individual image might exhibit features of several dermatological conditions, the stringent selection criteria required that included images showcased classic manifestations of their respective conditions, with the predominant representation being the condition of interest. Images that did not meet the criteria were excluded from the study. Inclusion in the final analysis required the unanimous agreement of both reviewers. Using this inclusion criteria, 54 images were selected for the final analysis, with 6 images selected for each of the 9 dermatological conditions.

### Clinical Scenario Creation

To provide a realistic assessment of GPT-4V’s capabilities in dermatological diagnosis and treatment planning, a set of 9 unique clinical scenarios was created. Each scenario corresponded to 1 of the 9 dermatological conditions under investigation. To ensure the accuracy and relevance of these scenarios, they were reviewed and approved by 2 board-certified dermatologists (JH and DT). Both reviewers, who are actively involved in medical education and have extensive experience with teaching clinical reasoning, contributed their expertise to the process. In cases where discrepancies arose between the reviewers, they were resolved through consensus discussions, ensuring the scenarios were both accurate and educationally appropriate. These scenarios represented the typical presentations of these conditions, encompassing common symptoms, patient history, and visual indicators (Supplemental Table 1). The clinical scenarios were specifically geared toward the medical student level.

### Diagnostic Evaluation

**Figure 1.**
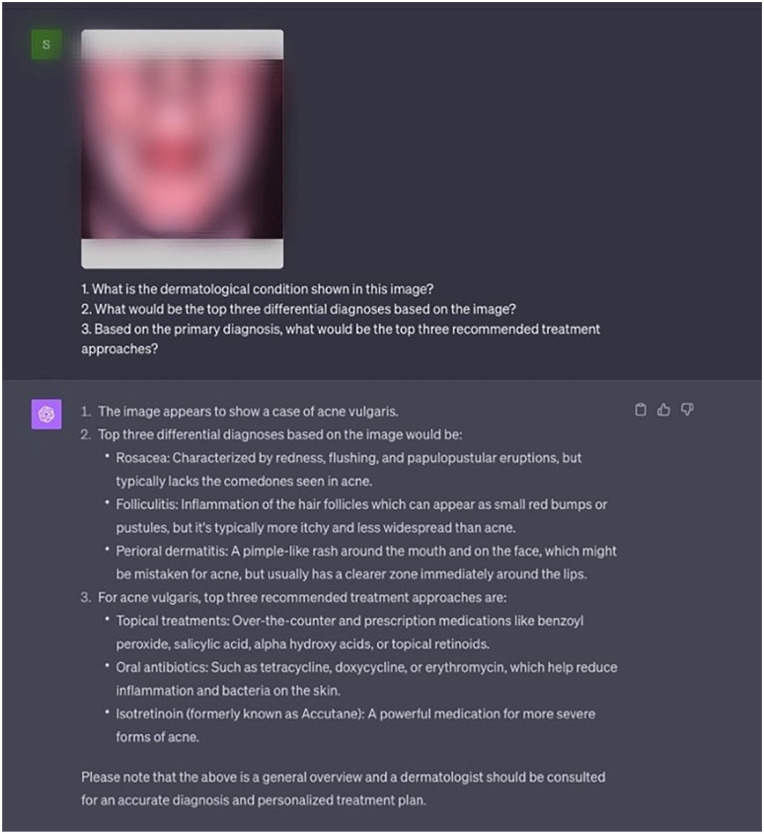
Example of the GPT-4V interface with the Image Prompt setup. Image blurred to respect copyright.

The evaluation of GPT-4V was conducted in 3 distinct setups using the ChatGPT platform, specifically, the version released on September 25, 2023. In each setup, 3 tailored prompts were provided to assess the model’s ability to provide accurate diagnoses and relevant treatment recommendations based on the input data (Supplemental Table 2). The prompts were constructed to reflect common clinical inquiries and were input into GPT-4V via the ChatGPT interface (Figure 1). The evaluation metrics were tailored to the 3 types of task prompts: diagnosis, differential diagnosis, and treatment recommendations (Supplemental Table 2).

In the Image Prompt setup, GPT-4V was provided with images depicting the dermatological condition. The prompts sought to evaluate its ability to identify the condition, propose differential diagnoses, and suggest treatment approaches solely based on visual input.

In the Scenario Prompt setup, textual descriptions of patient presentations were provided without a clinical image. The model was evaluated on its ability to diagnose conditions and suggest treatment approaches based on text input alone.

The Image + Scenario Prompt setup involved a multimodal input scenario where GPT-4V received both image data and textual descriptions of patient presentations. For this setup, each presentation was tested alongside a randomly selected image from the set corresponding to the same diagnosis. This aimed to evaluate the model’s ability to synthesize information from both text and image data to provide accurate diagnoses and treatment recommendations.

### Diagnostic Accuracy

The accuracy of diagnosis was assessed by comparing the model’s responses against the established ground truth. For the Image Prompt, the ground truth was ascertained from the database from which the images were sourced, alongside the verification by 2 board-certified dermatologists. For the Scenario Prompt, the ground truth was established based on the dermatologists’ (JH and DT) verification of the scenarios. The Image + Scenario setup utilized a combination of the above verification methods.

### Differential Diagnosis

The model’s efficacy in providing a differential diagnosis was evaluated by checking if the correct diagnosis was listed within the top 3 differential diagnoses generated by the model. This evaluation was consistent across all setups to assess the model’s ability to encompass a range of plausible conditions based on the provided input.

### Treatment Recommendations

Two dermatologists (JH and DT) evaluated the model’s treatment recommendations for the Scenario Prompt and Image + Scenario Prompt analyses using a modified version of an Entrustment Scale sourced from McMaster University’s “Competency by Design” model, which was adopted by the Royal College of Canada for residency education.^14^ Each correctly diagnosed outcome was rated on a scale from 1 to 5: (1) Model did not perform task or needed complete guidance; (2) Model required constant direction; (3) Model demonstrated some independence but required intermittent direction; (4) Independent but required supervision for safe practice and may have missed some nuances; and (5) Complete independence/understands risks/practice ready. The dermatologists independently rated the relevance and appropriateness of the treatment recommendations provided by the model for each condition. The evaluations from both dermatologists were then averaged to derive a consensus score. A Wilcoxon signed-rank test was performed to assess for any differences in performance in the Image + Scenario group versus the Scenario-only group.

## Results

### Image-Only Analysis

When assessing diagnostic accuracy with the Image-only inputs, GPT-4V correctly identified the primary diagnosis in 54% of the images and included the correct diagnosis in the differential list in 50% of the cases (Supplemental Table 3). Rosacea had the highest diagnostic accuracy, with the correct primary diagnosis given for all 6 images. AD and SCC had the lowest diagnostic accuracy, with the correct primary diagnosis provided for only 1 out of 6 images for each condition (Supplemental Table 4). When asked for the primary diagnosis, the model provided an answer for all 54 images with the exception of 1 image in the SSM group, where it described the characteristics of the lesion but failed to provide any diagnosis whatsoever. This was counted as incorrect in our analysis.

### Scenario-Only Analysis

When provided with Text-only scenarios, the model achieved 89% accuracy in identifying the primary diagnosis. The correct diagnosis was included in the list of differential diagnoses in 56% of cases (Supplemental Table 3). The model provided an incorrect primary diagnosis for only one of 9 cases (SCC) in the Scenario Prompt, although this was covered in the list of differential considerations.

### Image + Scenario Analysis

The multimodal analysis, which combines both an image and a clinical scenario, resulted in an 89% accuracy rate in identifying the primary diagnosis. The correct diagnosis was included in the list of differential diagnoses in 44% of the cases (Supplemental Table 3). The model provided an incorrect primary diagnosis for only one of 9 cases (SCC) in the Image + Scenario Prompt, and the differential diagnosis did not capture the correct condition. Considering the comparable performance of GPT-4V in the Scenario-only analysis and the multimodal analysis, further testing was conducted to determine whether the model is influenced by 1 modality over the other. The model was further evaluated on 2 unique cases to assess image or text preference: (1) A correctly diagnosed image presented with an incorrectly diagnosed clinical scenario, and (2) Two incorrectly diagnosed images presented with their correctly diagnosed clinical scenarios.

In the first case, an accurately diagnosed SCC image was combined with the incorrectly diagnosed clinical scenario. The results showed an incorrect primary diagnosis of the condition, without rectification in the list of the top 3 differential diagnoses.

In the second case, we evaluated the data from our previous multimodal input analysis and found 2 instances where an incorrectly diagnosed image, both in terms of primary and differential diagnoses, was presented with their correctly diagnosed clinical scenarios. This yielded a correct diagnosis of the primary condition in both cases. The list of differential diagnoses did not include the correct diagnosis.

### Entrustment Score Results

In the assessment of treatment recommendations, the average Entrustment Score was higher in the Image + Scenario group (4.067) compared to the Scenario-only (3.933) group. Entrustment Scores were not conducted for the Image-only assessments, given that coming up with a contextualized treatment plan is not feasible with only an image. Given the ordinal nature of the Entrustment Score data, a two-tailed Wilcoxon signed-rank test was performed. Given our sample size of n = 9 pairings, the *W*-value was used to evaluate the data instead of a *Z*-value. The value of *W* was 0, and the critical value for *W* at n = 9 (*P* < .01) was 1. There was thus a significant difference in performance by the model in providing treatment outcomes (*P* < .01) favoring the Image + Scenario group (Supplemental Table 3). The interrater agreement for this assessment was 37.5% between the raters involved in evaluating the treatment outcomes.

## Discussion

### Diagnostic Accuracy

The performance of GPT-4V in dermatology offers a multifaceted view into the capabilities and limitations of advanced language models in AI-driven diagnostics. The model’s 89% diagnostic accuracy for Text-only scenarios is perhaps unsurprising given ChatGPT’s performance on various standardized assessments.^11,15^ The observed diagnostic accuracy rate of 54% for Image-only inputs was underwhelming, especially given the extensive pretraining of GPT-4V on a vast corpus of internet images.^1,2^ However, pronounced variability in image-based diagnostic performance across different conditions was apparent, with high diagnostic accuracy for rosacea but low accuracy for AD and SCC (Supplemental Table 4). The model card explicitly reveals a lack of specialized training on medical images, which likely explains the model’s overall subpar image diagnostic accuracy performance.^1,2^ Together, the model’s underwhelming performance and a known lack of specialized training on medical images suggest that this GPT model was not trained on the images used in this study, although this cannot be verified with certainty. Moreover, OpenAI’s researchers pinpointed inconsistencies in medical imaging interpretation, underscoring a need for further alignment to improve medical diagnostic accuracy.^2^

A strategic approach of aligning and fine-tuning the model on domain-specific datasets could potentially bridge this accuracy gap. For instance, Med-Gemini, Google’s generalist multimodal medical model, was developed by fine-tuning and aligning the PaLM-E model to the biomedical domain.^16^ This process significantly enhanced its performance, achieving a Macro-AUC of 97.27% on the PAD-UFES-20, a dermatology dataset.^16^ Med-PaLM M’s performance and visual diagnostic capabilities suggest that improving GPT-4V’s diagnostic accuracy may require better alignment with the medical domain.

Across all 3 setups, the model performed worse overall in the differential diagnosis assessment as compared to the primary diagnosis assessment (Supplemental Table 3). This finding is perplexing, as the model would be expected to perform at least as well, if not better, in the differential diagnosis assessment. While the reason for this is unclear, this is an important area for improvement in addressing the model’s utility.

Our analysis suggests a tendency for the model to prioritize text over visual data, as observed where incorrect image diagnoses were corrected by textual diagnoses (Image + Scenario analysis). This was further evidenced when a correctly diagnosed image, paired with an incorrectly diagnosed clinical scenario, resulted in an overall incorrect diagnosis. The model would thus seem to prioritize textual information in both cases. The contrast between Text-only (89%) and Image-only (54%) performance (Supplemental Table 3) could suggest that the model has been reinforced to prioritize textual data, given its higher diagnostic accuracy in that category.

### Therapeutic Recommendations

Regarding treatment recommendations, a statistically significant difference (*P* < .01) in Entrustment Scores favored the Image + Scenario performance over the Scenario-only performance (Supplemental Table 3). This finding is peculiar as the opposite trend was observed in diagnostic evaluations, where the model performed better with textual or scenario-based data.

Feedback from 1 dermatologist (JH) likened the performance of GPT-4V to an early dermatology resident or an interested medical student, suggesting that the unaligned generalist model does have some promise to work alongside a doctor to assist, rather than to diagnose and treat. Augmenting the contextualization of images in relation to personalized therapeutic plans could further enhance the model’s performance.

Another dermatologist (DT) compared GPT-4’s treatment capabilities to those of a 4th-year medical resident, highlighting its proficiency in textbook treatments for various conditions. However, real-life clinical practice encompasses additional layers of decision-making that are crucial. For instance, in treating melanoma, GPT-4V tends to suggest advanced treatment options prematurely, overlooking essential steps such as initial biopsy, formal diagnosis, and staging. Similarly, while its approach to vitiligo treatment is technically accurate, it fails to account for the contextual and geographical nuances that significantly influence treatment decisions in real-life cases. These insights emphasize the important role of physicians in providing comprehensive care and highlight the necessity for enhanced contextual awareness in medical AI applications, extending beyond just textbook accuracy.^17^

### Study Limitations

The Entrustment Scale used to assess these treatment outcomes, despite offering a structured approach, has its limitations. The inherent subjectivity of the scale and its broad descriptors can lead to variability in evaluations, indicated by the interrater agreement score of 37.5%. In addition, this scale emphasizes the model’s independence in decision-making but may not accurately reflect the effectiveness or appropriateness of the proposed treatments. The risk of overestimating the AI’s capabilities through this scale suggests a need for more nuanced and context-specific assessment methods to appropriately evaluate such models in future studies. While the Entrustment Score analysis was statistically significant, our smaller sample size of 9 pairings introduces the risk of statistical bias and effect size inflation.

The study members (JH and DT) who developed the clinical scenarios were also responsible for evaluating the AI’s performance in those scenarios. This overlap could introduce potential bias in how the AI’s performance might be assessed based on expectations.

### Limitations and Biases of AI in Dermatology

Despite considerable potential, the integration of LLMs into dermatology risks reinforcing long-standing racial biases and disparities. Dermatology’s history of focusing on white skin tones in research and educational resources has led to a significant underrepresentation of richly pigmented skin.^18,19^ Consequently, AI models, often trained on datasets dominated by images of patients with white skin, may inadvertently perpetuate these disparities. A 2018 study demonstrated the superior performance of deep-learning convolutional neural networks in identifying potentially cancerous skin lesions compared to most dermatologists.^20^ However, the study used images primarily from the International Skin Imaging Collaboration: Melanoma Project, which predominantly features dermatological conditions on white skin from populations in the USA, Europe, and Australia.^20,21^ This bias in AI training can result in less accurate diagnoses or delayed recognition of skin conditions in patients with darker skin tones, exacerbating disparities in outcomes.^17-19,21^ These are compounded by data security and privacy concerns associated with AI.^22^

As LLMs become more integrated within medicine, their potential to impact the quality of care grows.^23,24^ This underscores the urgent need for comprehensive regulatory frameworks. Such frameworks should mandate rigorous testing of these AI tools to identify and mitigate biases across different skin types, ensuring they adhere to stringent standards of accuracy and fairness. Moreover, dermatologists and healthcare professionals employing these AI tools must be cognizant of their inherent limitations and biases. It is imperative that clinicians using AI tools maintain a high level of clinical judgment, particularly when treating patients from diverse racial backgrounds. This approach will help ensure that the deployment of LLMs in dermatology contributes positively to patient care, respecting the nuances of diverse skin types and promoting equitable healthcare practices.

## Conclusion

GPT-4V has the potential to enhance dermatological diagnostics and treatment planning, yet its performance highlights significant areas requiring refinement in both image-based and multimodal analyses. This study explores the limitations of commercially available AI models such as GPT-4V and pinpoints areas for improvement in multimodal integration and contextual reasoning within clinical dermatology. Addressing these gaps through targeted refinements and domain-specific training could enhance GPT-4V’s potential to augment dermatological practice.

## Supplemental Material

sj-docx-1-cms-10.1177_12034754251336238 – Supplemental material for Evaluating the Diagnostic and Treatment Capabilities of GPT-4 Vision in Dermatology: A Pilot Study Supplemental material, sj-docx-1-cms-10.1177_12034754251336238 for Evaluating the Diagnostic and Treatment Capabilities of GPT-4 Vision in Dermatology: A Pilot Study by Abhinav Pillai, Sharon Parappally-Joseph, Jason Kreutz, Danya Traboulsi, Maharshi Gandhi and Jori Hardin in Journal of Cutaneous Medicine and Surgery

## References

[1] Yenduri G Ramalingam M Selvi GC , et al GPT (generative pre-trained transformer)—a comprehensive review on enabling technologies, potential applications, emerging challenges, and future directions. IEEE Access. 2024;12:54608-54649. doi:10.1109/access.2024.3389497

[2] OpenAI. GPT-4V(ision) System Card. September 25, 2023. Accessed November 4, 2023. https://cdn.openai.com/papers/GPTV_System_Card.pdf

[3] Gabashvili IS . ChatGPT in dermatology: a comprehensive systematic review. Published online June 12, 2023. doi:10.1101/2023.06.11.23291252

[4] Kluger N . Potential applications of ChatGPT in dermatology. J Eur Acad Dermatol Venereol. 2023;37(7):e941-e942. doi:10.1111/jdv.19152 37102458

[5] Saeed M Naseer A Masood H Rehman SU Gruhn V . The power of generative AI to augment for enhanced skin cancer classification: a deep learning approach. IEEE Access. 2023;11:130330-130344.

[6] O’Hern K Yang E Vidal NY . ChatGPT underperforms in triaging appropriate use of Mohs surgery for cutaneous neoplasms. JAAD Int. 2023;12:168-170. doi:10.1016/j.jdin.2023.06.002 37404248 PMC10316650

[7] Shapiro J Lyakhovitsky A . Revolutionizing teledermatology: exploring the integration of artificial intelligence, including generative pre-trained transformer chatbots for artificial intelligence-driven anamnesis, diagnosis, and treatment plans. Clin Dermatol. 2024;42(5):492-497. doi:10.1016/j.clindermatol.2024.06.020 38942153

[8] Cazzato G Rongioletti F . Artificial intelligence in dermatopathology: updates, strengths, and challenges. Clin Dermatol. 2024;42(5):437-442. doi:10.1016/j.clindermatol.2024.06.010 38909860

[9] Luo N Zhong X Su L Cheng Z Ma W Hao P . Artificial intelligence-assisted dermatology diagnosis: from unimodal to multimodal. Comput Biol Med. 2023;165:107413. doi:10.1016/j.compbiomed.2023.107413 37703714

[10] Wongvibulsin S Yan MJ Pahalyants V Murphy W Daneshjou R Rotemberg V . Current state of dermatology mobile applications with artificial intelligence features. JAMA Dermatol. 2024;160(6):646-650. doi:10.1001/jamadermatol.2024.0468 38452263 PMC10921342

[11] Brin D Sorin V Vaid A , et al Comparing ChatGPT and GPT-4 performance in USMLE soft skill assessments. Sci Rep. 2023;13(1):16492. doi:10.1038/s41598-023-43436-9 37779171 PMC10543445

[12] Lee P Bubeck S Petro J . Benefits, limits, and risks of GPT-4 as an AI chatbot for medicine. N Engl J Med. 2023;388(13):1233-1239. doi:10.1056/NEJMsr2214184 36988602

[13] Ferreira AL Chu B Grant-Kels JM Ogunleye T Lipoff JB . Evaluation of ChatGPT dermatology responses to common patient queries. JMIR Dermatol. 2023;6:e49280. doi:10.2196/49280 PMC10692871 37976093

[14] McMaster University Postgraduate Medical Education Office. CBME-Talk-the-Talk. June. 2020. Accessed November 4, 2023. https://pgme.mcmaster.ca/app/uploads/2020/06/CBME-Talk-the-Talk.pdf

[15] Goktas P Grzybowski A . Assessing the impact of ChatGPT in dermatology: a comprehensive rapid review. J Clin Med. 2024;13(19):5909. doi:10.3390/jcm13195909 39407969 PMC11477344

[16] Tu T Azizi S Driess D , et al Towards generalist biomedical AI. NEJM AI. 2024;1(3). doi:10.1056/aioa2300138

[17] Wei ML Tada M So A Torres R . Artificial intelligence and skin cancer. Front Med (Lausanne). 2024;11:1331895. doi:10.3389/fmed.2024.1331895 38566925 PMC10985205

[18] Kamath P Sundaram N Morillo-Hernandez C Barry F James AJ . Visual racism in internet searches and dermatology textbooks. J Am Acad Dermatol. 2021;85(5):1348-1349. doi:10.1016/j.jaad.2020.10.072 33130182

[19] Narla S Heath CR Alexis A Silverberg JI . Racial disparities in dermatology. Arch Dermatol Res. 2023;315(5):1215-1223. doi:10.1007/s00403-022-02507-z 36508020 PMC9743121

[20] Haenssle HA Fink C Schneiderbauer R , et al Man against machine: diagnostic performance of a deep learning convolutional neural network for dermoscopic melanoma recognition in comparison to 58 dermatologists. Ann Oncol. 2018;29(8):1836-1842. doi:10.1093/annonc/mdy166 29846502

[21] Adamson AS Smith A . Machine learning and health care disparities in dermatology. JAMA Dermatol. 2018;154(11):1247-1248. doi:10.1001/jamadermatol.2018.2348 30073260

[22] Gordon ER Trager MH Kontos D , et al Ethical considerations for artificial intelligence in dermatology: a scoping review. Br J Dermatol. 2024;190(6):789-797. doi:10.1093/bjd/ljae040 38330217

[23] De A Sarda A Gupta S Das S . Use of artificial intelligence in dermatology. Indian J Dermatol. 2020;65(5):352-357. doi:10.4103/ijd.IJD_418_20 33165383 PMC7640800

[24] Omiye JA Gui H Daneshjou R Cai ZR Muralidharan V . Principles, applications, and future of artificial intelligence in dermatology. Front Med (Lausanne). 2023;10:1278232. doi:10.3389/fmed.2023.1278232 37901399 PMC10602645

